# *In silico* identification of *Capsicum* type III polyketide synthase genes and expression patterns in *Capsicum annuum*

**DOI:** 10.1515/biol-2020-0077

**Published:** 2020-10-12

**Authors:** Delong Kan, Di Zhao, Pengfei Duan

**Affiliations:** Henan Provincial Academician Workstation of Water Security for Water Source Region of Mid-line of South-to-North Diversion Project, Collaborative Innovation Center of Water Security for Water Source Region of Mid-line of South-to-North Diversion Project of Henan Province, Henan Provincial Key Laboratory of Ecological Security for Water Source Region of Mid-line of South-to-North Diversion Project, Nanyang Normal University, Nanyang, Henan Province, 473061, China; College of Environmental Science and Tourism, Nanyang Normal University, Nanyang, Henan Province, 473061, China

**Keywords:** type III polyketide synthase, chalcone synthase, *Capsicum*, flavonoids

## Abstract

Studies have shown that abundant and various flavonoids accumulate in chili pepper (*Capsicum*), but there are few reports on the genes that govern chili pepper flavonoid biosynthesis. Here, we report the comprehensive identification of genes encoding type III polyketide synthase (PKS), an important enzyme catalyzing the generation of flavonoid backbones. In total, 13, 14 and 13 type III PKS genes were identified in each genome of *C. annuum*, *C. chinense* and *C. baccatum*, respectively. The phylogeny topology of *Capsicum* PKSs is similar to those in other plants, as it showed two classes of genes. Within each class, clades can be further identified. Class II genes likely encode chalcone synthase (CHS) as they are placed together with the *Arabidopsis* CHS gene, which experienced extensive expansions in the genomes of *Capsicum*. Interestingly, 8 of the 11 Class II genes form three clusters in the genome of *C. annuum*, which is likely the result of tandem duplication events. Four genes are not expressed in the tissues of *C. annuum*, three of which are located in the clusters, indicating that a portion of genes was pseudogenized after tandem duplications. Expression of two Class I genes was complementary to each other, and all the genes in Class II were not expressed in roots of *C. annuum*. Two Class II genes (CA00g90790 and CA05g17060) showed upregulated expression as the chili pepper leaves matured, and two Class II genes (CA05g17060 and CA12g20070) showed downregulated expression with the maturation of fruits, consistent with flavonoid accumulation trends in chili pepper as reported previously. The identified genes, sequences, phylogeny and expression information collected in this article lay the groundwork for future studies on the molecular mechanisms of chili pepper flavonoid metabolism.

## 1 Introduction

Type III polyketide synthases (PKSs) play important roles in plant secondary metabolism by generating backbones of a variety of flavonoids such as anthocyanins, chalcones, aurones, stilbenes, pyrones and benzophenones [1,2,3,4,5]. Despite being functionally similar, they are structurally and mechanistically distinct from bacterial type I and type II PKSs. A typical type III PKS forms a homodimer of peptides with a size of 40–45 kDa and catalyzes polyketide assembly by sequential decarboxylation of malonyl-CoA, resulting in chain elongation of a CoA-linked starter substrate [6,7]. Type III PKSs utilize various CoA-linked starter substrates, and the chain elongation reactions usually involve one to three molecules of malonyl-CoA, although some elongation can condense up to eight steps [4]. The elongation products usually serve as linear intermediates which are further cyclized to generate polyketide scaffolds with different structures, while a small portion of type III PKSs, benzalacetone synthase and curcuminoid synthase, only function by condensation without cyclization [8,9,10]. Crystallographic and structure-based mutagenesis studies have revealed structural details of the type III PKS-catalyzed reactions, which show that the enzymes share a common three-dimensional fold with a conserved Cys-His-Asn catalytic triad in an internal active site as a key property of the functionally active enzymes, and the shape of the conserved cavity governs the starter molecule selectivity, the polyketide chain length and the cyclization reactions [11,12,13].

Among plant type III PKSs, chalcone synthase (CHS) is a common and well-studied enzyme, which has been demonstrated to be ubiquitous in plants [14]. Using three molecules of malonyl-CoA, it catalyzes iterative decarboxylative condensation of pcoumaroyl-CoA to produce a new aromatic ring system, naringenin chalcone, serving as the key intermediate in the biosynthesis of flavonoids, playing important roles in flower plants [4]. It is the first among the type III PKSs isolated and the simplest representative of the type III PKS family [15]. Like other type III PKSs, CHSs often exist as small-to-large families in flower plants, resulting from segmental or whole-genome duplication and nucleotide substitution [16,17]. They are constitutively expressed among tissues and across different developmental stages [18]; and they are induced by external factors such as drought, salinity, low temperature, UV, wounding and biotic infections [19,20,21,22,23,]. Plant CHSs play significant roles in physiological, pathological and developmental processes such as flower pigmentation, fruit development and biotic and abiotic resistance [23,24,25,26]. Since the first discovery of CHSs in parsley in 1983, nearly 20,000 plant CHSs have been cloned (reported in NCBI as on 8 February 2020), and extensive studies have been carried out on the evolution, expression and functions of CHS genes in plants [1,14].

Chili pepper is grown and consumed all over the world as a fresh vegetable or as a dried spice. It is used in medicine and also in the perfume industry. Studies have shown that flavonoids are present in the fruits and leaves of chili pepper, probably conferring pepper plants defense against abiotic and biotic stress, serving as development regulators of auxin transporters, and have the potential of being utilized by humans as bioactive compounds [27,28,29]. Variations have been discovered between tissues and developmental stages in chili peppers. For example, Lightbourn et al. (2007) showed that anthocyanin levels were higher in mature leaves as compared to those in immature ones [30]. Marin et al. (2004) demonstrated that flavonoid levels were the highest in the immature state of fruit and decreased as the fruits matured [31]. Additionally, Howard et al. (2000) showed variations across fruit developmental stages in chili pepper [32]. Despite the significant roles flavonoids play in the physiology of chili peppers, few studies have focused on the molecular aspects of flavonoid biosynthesis in *Capsicum*. It is reasonable to propose that type III PKSs are important in regulating flavonoid biosynthesis in chili peppers, as demonstrated in other plants [1,14]. In this article, we report comprehensive identification of type III PKS genes in the genomes of three *Capsicum* species and survey gene expression in *Capsicum annuum*.

## 2 Materials and methods

### 2.1 Chili pepper seed germination and growth conditions

The chili pepper seeds (Chaola No. 3) were obtained from Yalong Seed Limited, Suzhou, Anhui, China. The seeds were then treated with hot water (55°C) for 20 min and soaked in water for 5 h at room temperature before they were put into a dark growth chamber and covered with wet towels. When seeds started to germinate, they were transferred to pots in a greenhouse. Thirty days after germination (DAG), plants were transferred from pots to the experimental field of Nanyang Normal University in Nanyang, Henan Province, China. Healthy leaves without damage were collected at 20, 40 and 60 DAG. Young (1 cm), green mature and red mature fruits were collected.

### 2.2 RNA isolation and quantitative reverse transcription-PCR (qRT-PCR) analysis

Total RNA was extracted from the collected samples using TRIzol reagent and treated with DNase I to remove genomic DNA contamination. First-strand cDNA was synthesized using SuperScript II reverse transcriptase (Invitrogen) with 3  μg of total RNA as a template for each sample. qPCR was performed as per the following protocol: 10 μL of 2× SYBR Green PCR Master Mix, 1 μL of gene-specific primers (2.0 μM) and 2 μL of cDNA (10-fold dilution), adding RNase-free water to a final volume of 20 µL. ABI 7500 Real-Time PCR System (Applied Biosystems, USA) was used for qPCR using the following thermal cycling conditions: 95°C for 2 min; 25 cycles of 95°C for 20 s, 55°C for 30 s and 72°C for 31 s. The primers for qPCR are listed in Table 1. Alpha-tubulin was used as a reference sequence for quantification. The specificity of primer pairs was checked by melting-curve analysis. For each gene, the lowest detectable expression level was assumed to be 100% transcription abundance. The PCR reactions were performed in three biological replicates for each sample. The resulting data are presented as mean ± SD (*n* = 3). Significance testing was performed using the command “*t*-test” in R (https://www.r-project.org/).

**Table 1:** Primers used for qPCR

ID	Left	Right
CA03g02050	CTGCAGTCACATTTCGTGGG	TGCTGAGACGAGCTGGAATAAA
CA12g20050	AGGTTGCTTTGGTGGTGGT	CACTTGGGCCATGGAAGGT
CA12g20060	ACAGCCATTCCTCTTAATTGTGTTG	ACACATGCGCTTAAACTTTGCT
CA12g20070	GGTGGTGGCGCTGTTCT	CACTTGGGCCATGGAAGGT
CA08g18780	AGGCCACTTTCAGACATTACACA	CTCCTCCAGAACAACCAGCAA
CA05g17060	TTTCTGCGGCCCAAACTCT	GCTTCTATCAAACTCTTCTCGATATTCTT
CA00g32570	TTCCTTCACAACTCGTCCCTC	GTGTATCTTGTCTTCACAGTAGTAGT
CA00g90790	GCGATCGTTCAAGTGCCAA	GTGAGTTGATAGTCCGCCCC
CA00g90800	GCGTTGTTCAGTGATGGGG	GCTATTTGGGAGAAGAGTTTGAGTT

### 2.3 Identification of type III PKS sequences

The genomes of *Capsicum annuum*, *C. chinense*, *C. baccatum*, *Nicotiana sylvestris*, *Petunia inflata* and *Solanum lycopersicum* (tomato) were used for gene identification [33,34,35,36,37]. Genomes of three *Capsicum* species are publicly available, which were all used for gene type III PKS identification. Tomato (*Solanum lycopersicum*) is a well-known crop, *Nicotiana sylvestris* is a diploid *Nicotiana* species and *Petunia inflata* is well known for anthocyanin accumulation in flowers. The aforementioned species represent major clades in the evolution of the family Solanaceae. Two conserved domains, PF00195.18 and PF02797.14, were identified in *Arabidopsis* CHS peptides (AT5G13930.1) by querying the Pfam database [38]. Peptide sequences with the two domains were mined using HMMER-based search with default parameters [39].

### 2.4 Sequence alignment, phylogenetic analysis and chromosomal localization of the genes

Global alignment of the peptide sequences was performed using MUSCLE v3.8.31 [40]. Phylogenetic analysis was conducted with PhyML 3.0 with 100 bootstrap replicates [41]. Based on the GFF3 files provided by the authors of the *C. annuum* genome assembly [33], the chromosomal locations of the genes were drawn using MapChart 2.30 [42].

### 2.5 *In silico* characterization of gene expression

Transcriptome data were downloaded from NCBI, which were produced by the authors of the *C. annuum* genome (accession ID: PRJNA223222) [33]. Reads were mapped to the *C. annuum* genome, and FPKM values were calculated using Kallisto v0.45 [43]. Heat maps were drawn by ggplot2, an R package [44].

## 3 Results

### 3.1 Identification and phylogeny of type III PKS sequences

With the availability of genomes for three *Capsicum* species [33,34], we mined type III PKS sequences using the two conserved domains as queries (PF00195.18 and PF02797.14). In total, 13, 14 and 13 genes were identified in each of the three *Capsicum* genomes (*Capsicum annuum*, *C. chinense* and *C. baccatum*, respectively). In addition, 7, 6 and 9 genes were identified in the genomes of tomato, *Nicotiana sylvestris* and *Petunia inflata*, respectively, which belong to Solanaceae as *Capsicum* do.

**Figure 1:**
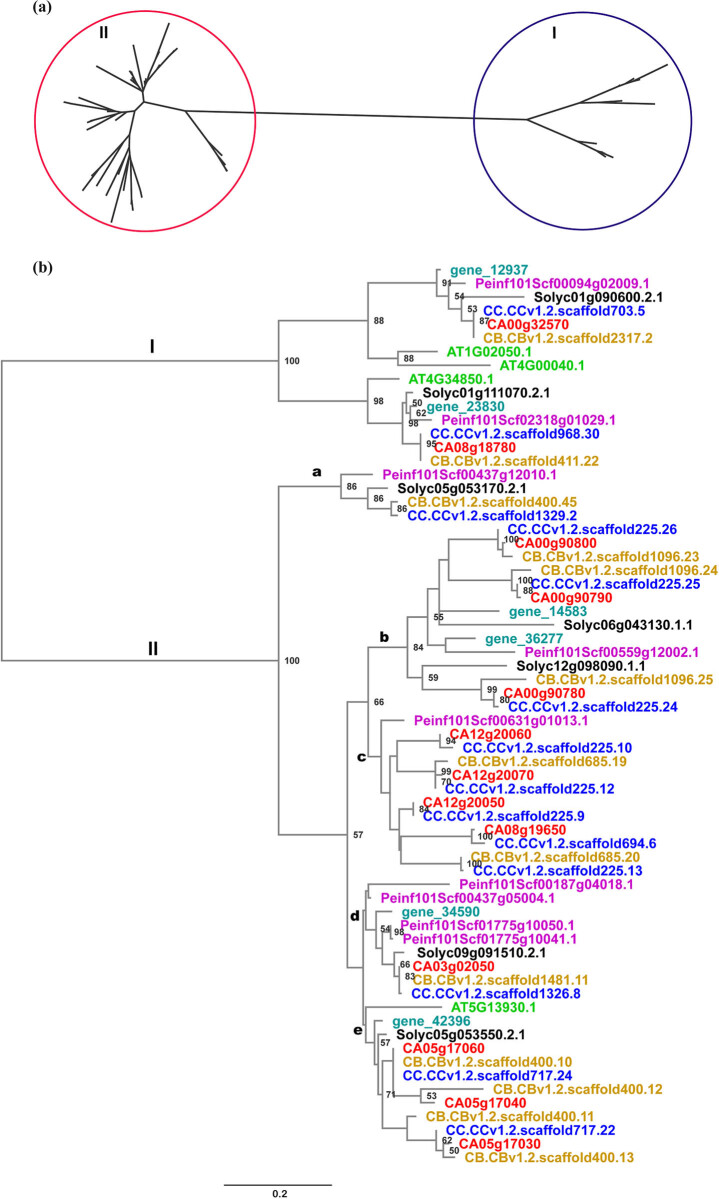
Phylogenetic relationships of PKSs. (a) Unrooted phylogenetic tree of PKSs, which clearly shows that the genes are split into two major clades represented by I and II. (b) Phylogenetic tree of PKSs rooted with the branch linking the two major clades as suggested in (a). In addition to the two classes labeled as in (a), the five clades are labeled as a–e. Shown at nodes are bootstrap values based on 100 resampling replicates. Tips with different colors represent gene IDs from different species, whose color codes are as follows: red, *Capsicum annuum*; blue, *Capsicum chinense*; brown, *Capsicum baccatum*; black, *Solanum lycopersicum*; light blue, *Nicotiana sylvestris*; pink, *Petunia inflata*; and green, *Arabidopsis thaliana*.

The type III PKS peptide sequences were globally aligned, and the phylogenetic relationships were resolved with *Arabidopsis* homologs as references using PhyML under the maximum likelihood criterion. As expected, the type III PKS peptides were clustered into two major groups, designated as Class I and Class II (Figure 1) [45]. The Class I peptides are further divided into two clades, with one in each clade corresponding to each *Capsicum* species, clustering with *Arabidopsis* PKSA (AT1G20250.1) and PKSB (AT4G34850.1), respectively [46,47]. The *Arabidopsis CHS* gene is in Class II, suggesting that the genes in this group function as CHS. The Class II peptides are further clustered into at least five clades, labeled as a–e (Figure 1 and Table A1). Among the *C. annuum* genes, none is present in Clade a, while there are 3, 4, 1 and 3 genes in Clade b, c, d and e. A tomato and a tobacco gene were clustered together within the same clade as the *Arabidopsis* CHS (AT5G13930.1); in this clade, there are three genes in *C. annuum*, four in *C. baccatum* and two in *C. chinense*. In the other four clades, no *Arabidopsis* representatives are present, indicating that the CHS homologs in Class II have experienced several rounds of duplication in Solanaceae. Overall, we have observed at least three *Capsicum*-specific expansion events in this class (Figure 1).

### 3.2 Chromosomal location and tandem duplication of type III PKSs in *C. annuum*

**Figure 2:**
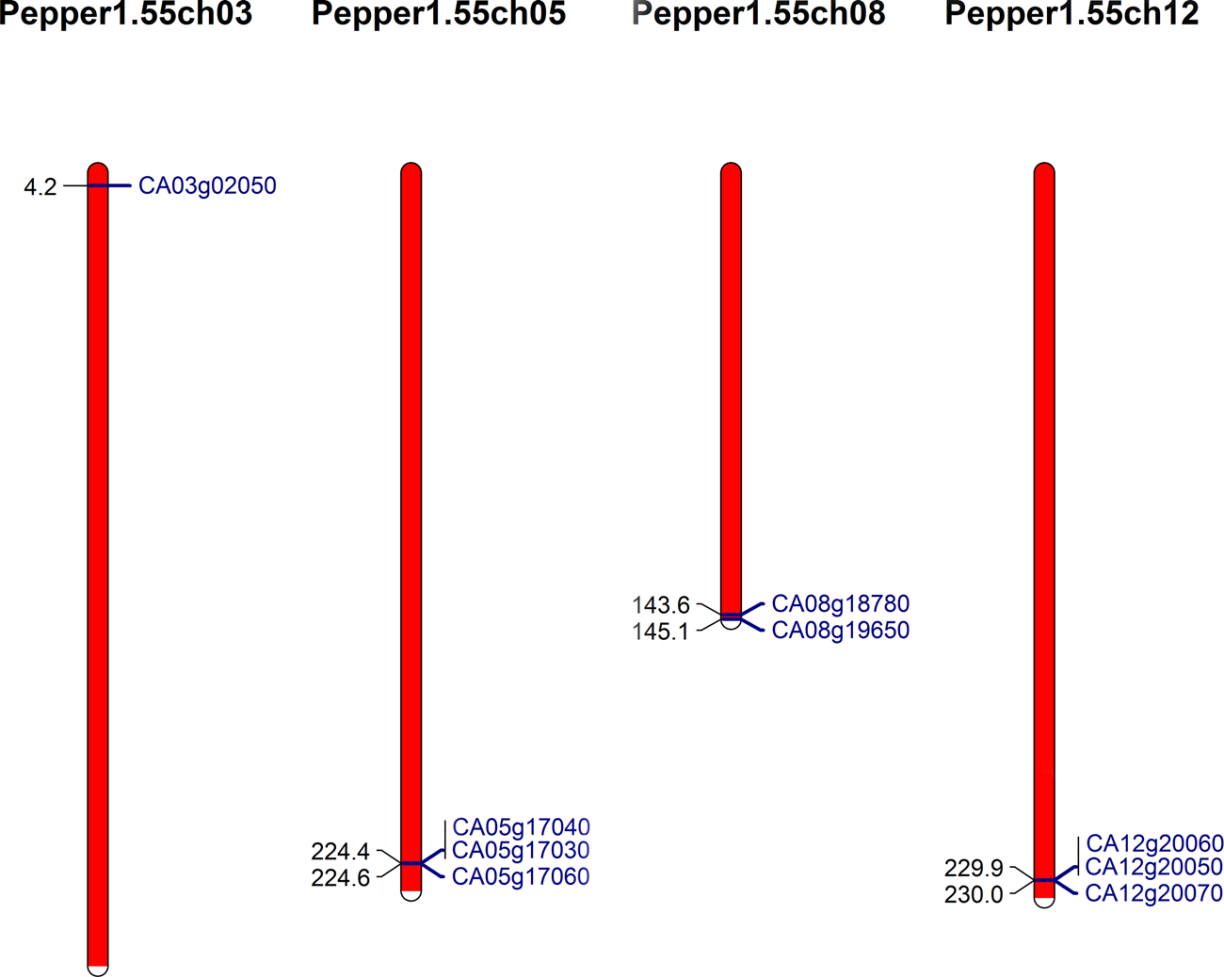
Chromosome map of type III PKS genes in chili pepper (*Capsicum annuum*).

Of the three *Capsicum* species with assembled genomes, the species *C. annuum* is the most widely cultivated around the globe, and its genome assembly is only anchored to pseudomolecules (chromosomes) [33]. Here, we further characterize the chromosomal location of type III PKS genes in *C. annuum*. Of the 13 type III PKS genes, 9 are located on the pseudomolecules, which are in chr. 3, 5, 8 and 12 (Figure 2), and the other 4 are located in contigs that are not anchored to chromosomes. Interestingly, there are three groups of genes demonstrating clusters. The first one is in chr. 5 (CA05g17030, CA05g17040 and CA05g17060), the second one is in chr. 12 (CA12g20050, CA12g20060 and CA12g20070) and the third one is in a scaffold not anchored to pseudomolecules (CA00g90780, CA00g90790 and CA00g90800). All the clustered genes are in the Class II (CHS) group; 9 of the 11 Class II genes are clustered together, accounting for 82% of the chili pepper Class II genes, indicating that CHS gene expansion events in chili pepper are mainly due to tandem duplications.

### 3.3 Type III PKS gene expression among *C. annuum* tissues

With the availability of RNASeq data, we have analyzed gene expression in different tissues of *C. annuum*. Different expression patterns are demonstrated between the two classes of genes. Two Class I genes show expression complementary to each other, with CA00g32570 expressed in leaves, while CA08g18780 expressed in most of the other tissues. There are four Class II genes (CA00g90780, CA08g19650, CA05g17040 and CA05g17030) without RNASeq mapped reads, suggesting that they are not expressed in *C. annuum* tissues. Considering the large number of Class II genes, the above results indicate that some genes have been pseudogenized after an extensive expansion of Class II genes in *C. annuum*.

**Figure 3:**
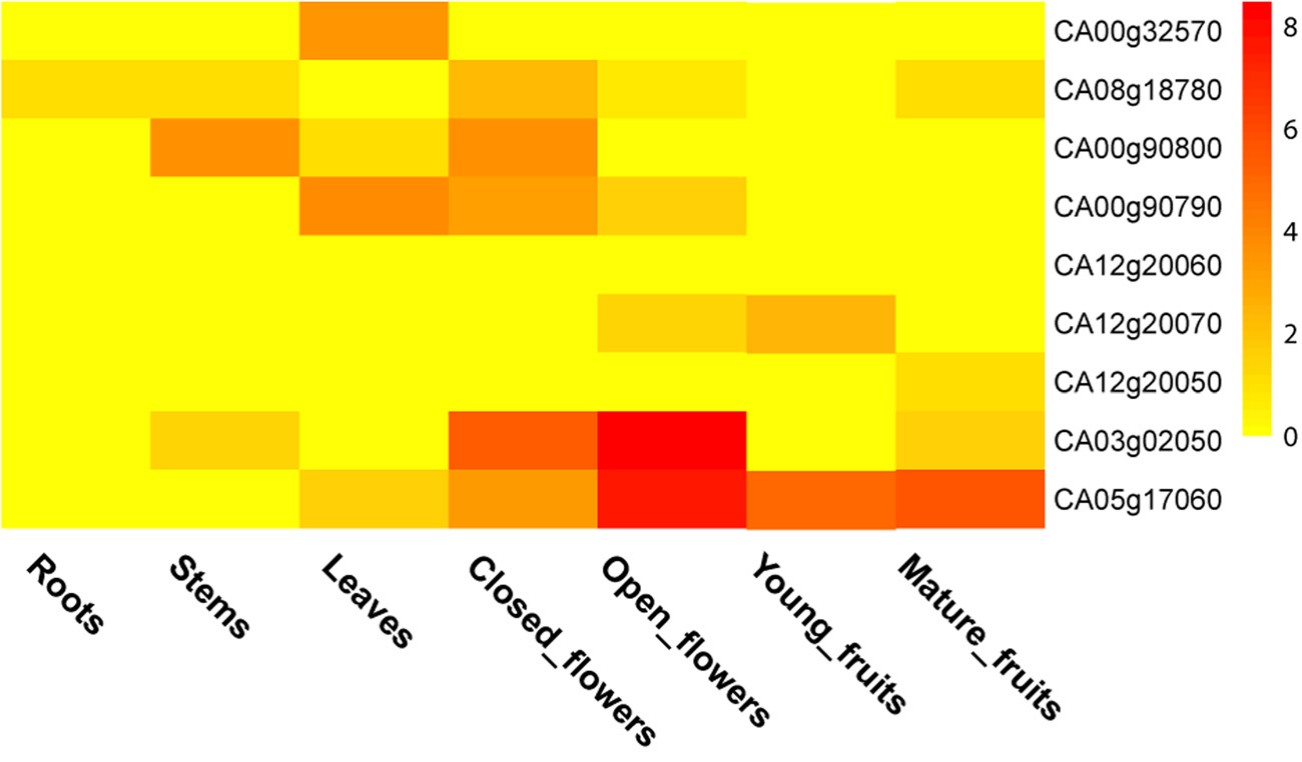
Expression of type III PKSs in different tissues of chili pepper (*Capsicum annuum*). The raw read data were downloaded from NCBI SRA database. The spectrum from yellow to red represents log2-transformed FPKM values.

Among tissues, different numbers of genes are expressed. In roots, all the genes are not expressed or expressed at very low levels. In stems and leaves, reads for three (CA08g18780, CA00g90800 and CA03g02050) and four genes (CA00g32570, CA00g90800, CA00g90790 and CA05g17060) were detected, respectively. In fruits, the two Class I genes (CA00g32570 and CA08g18780) are not expressed or expression occurs at very low levels, while the genes in Clade d (CA03g02050) and e (CA05g17060) in Class II are highly expressed (Figure 3).

### 3.4 Expression of type III PKS genes across developmental stages of leaves and fruits in *C. annuum*

**Figure 4:**
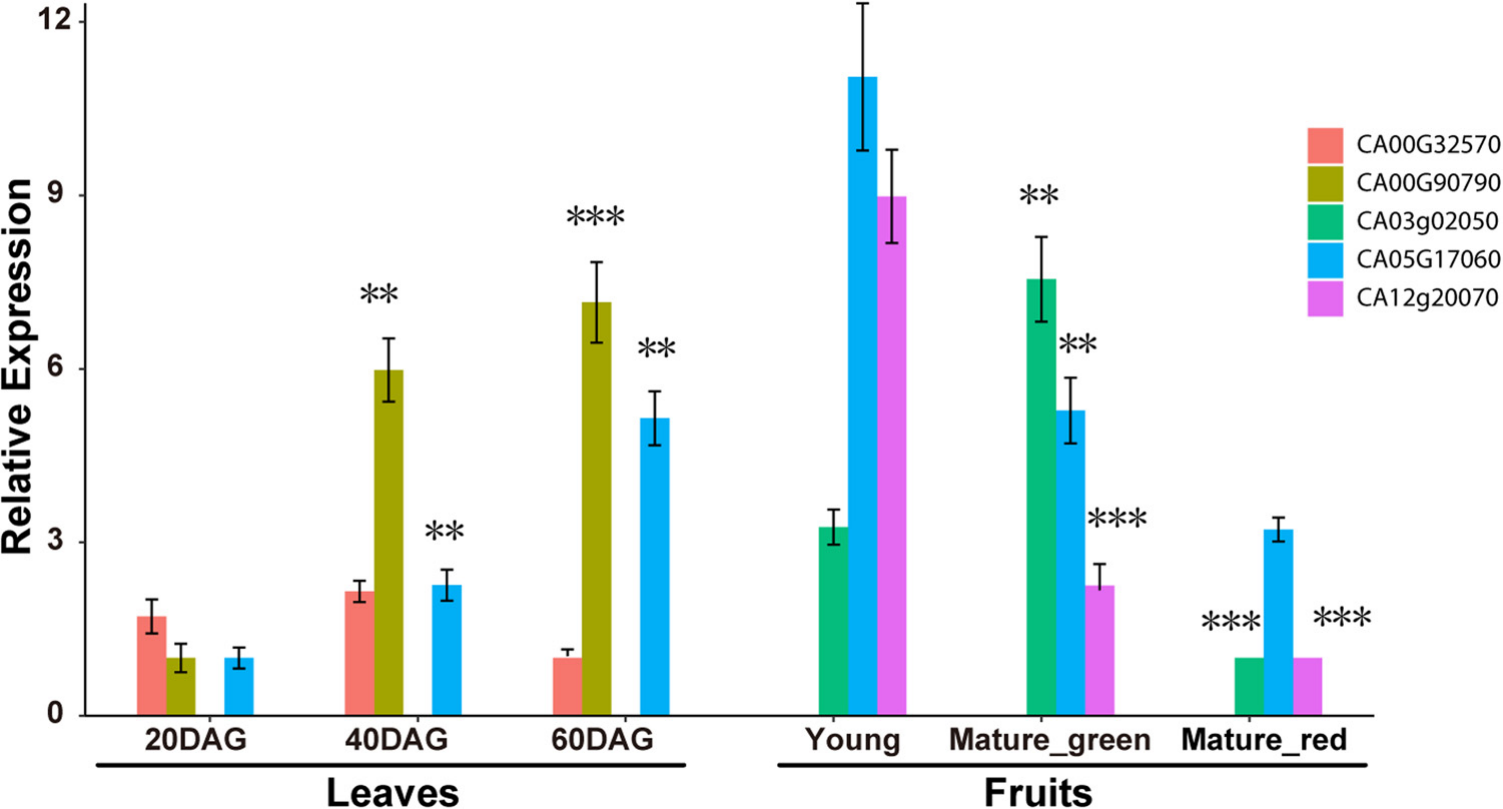
Expression of type III PKS genes in different developmental stages of chili pepper leaves and fruits calculated from qRT-PCR results. Shown at *y*-axis are mean of expression values of three independent experiments, and error bars represent standard deviations. *P* values of 0.001–0.01 were marked with two asterisks, and *P* values less than 0.001 were marked with three asterisks.

Previous articles have revealed differences in the flavonoid content across the developmental stages of chili pepper leaves and fruits [30,31]. To postulate the relationship between type III PKS gene expression and flavonoid biosynthesis, we have gauged the expression of type III PKS genes by qRT-PCR in chili pepper leaves and fruits across different developmental stages. Of the nine genes that had mapped RNASeq reads, only five genes had detectable expression in the collected samples corresponding to the three stages of leaf development and three fruit developmental stages. In leaves, expression of one Class I gene (CA00g32570) and two Class II genes (CA00g90790 and CA05g17060) was detected. At 20 DAG, the three genes were expressed at low levels, while in 40 and 60 DAG, expression of the two Class II genes was significantly upregulated. At 40 and 60 DAG, the expression of the gene CA00g90790 was elevated 4.98- (*P* = 0.002, Student’s *t*-test) and 6.15-fold (*P* < 0.001, Student’s *t*-test), respectively, and that of CA05g17060 increased 1.26- (*P* = 0.004, Student’s *t*-test) and 4.15-fold (*P* = 0.001, Student’s *t*-test), respectively. However, the expression change of the Class I gene was not significant at the two sampling points (both *P* > 0.05, Student’s *t*-test). The upward trend of expression of the two *CHS* genes during the leaf development is consistent with the pattern of flavonoid accumulation across the leaf developmental stages in chili pepper [30]. In the chili pepper fruit, no Class I gene expression was detected, while the expression of three Class II genes (CA03g02050, CA05g17060 and CA12g20070) was detected. The expression of CA03g02050 was highest in mature green fruits, with expression levels 2.2-fold higher as compared to young fruits (*P* < 0.001, Student’s *t*-test), and decreased 6.55-fold in mature fruits (*P* < 0.001, Student’s *t*-test). The expression of CA05g17060 and CA12g20070 was highest in young fruits. In mature green and mature fruits, the expression of CA05g17060 was decreased to 47.8% (*P* = 0.006, Student’s *t*-test) and 29.1% (*P* = 0.002, Student’s *t*-test) of that in young fruits, respectively, and the expression of CA12g20070 was downregulated to 25.1% and 11.1% (both *P* < 0.004, Student’s *t*-test) of that in young fruits, respectively, consistent with the accumulation mode of flavonoids in chili pepper fruits (Figure 4) [31].

## 4 Discussion

Flavonoids constitute the largest subclass of phenolics in plants, with over 10,000 structures discovered to date. They play important roles in a wide spectrum of processes of plant development, physiology and pathology [1,2,6,40]. As in other plants, there are a wide variety of flavonoids in chili pepper, which have been linked with various physiological and developmental processes. Although molecular mechanisms of flavonoid biosynthesis have been extensively studied in many plants, few studies have been performed in *Capsicum*, an important vegetable. With the availability of published genomes, we have carried out comprehensive mining of the type III PKS genes in three *Capsicum* species [20,21]. This gene has been extensively studied, which has been revealed to catalyze the committed step of flavonoid biosynthesis in a wide variety of plants and other organisms [14,42].

A total of 13, 14 and 13 type III PKS genes were identified in the genomes of *C. annuum*, *C. chinense* and *C. baccatum*, respectively, which can be classified into two classes. The *Arabidopsis* CHS gene is clustered in Class II, indicating that Class II genes encode CHS in *Capsicum* and other Solanaceae species. Most genes were found close to each other in the phylogenetic tree, indicating that the genes are conserved within the genus of *Capsicum*. The genes have experienced expansions both in Solanaceae and in *Capsicum*. For example, in Class II, there is only 1 gene in *Arabidopsis*, but there are 5 genes in tomato, and there are 11 in each *Capsicum* species, which indicates that CHS plays important roles in Solanaceae, especially in *Capsicum*.

CHS genes have likely experienced tandem duplications in *Capsicum*, forming three clusters in the *Capsicum annuum* genome. It will be interesting to identify the roles of CHS in *Capsicum*, which might be very important in the species, as it has been reported that tandem duplication of a gene may result in a significant accumulation of particular specialized metabolites, as demonstrated in *Capsicum* and coffee [33,48]. Four genes, likely pseudogenized, were all in Class II which has experienced extensive expansion. Pseudogenization occurred frequently in other gene families which have experienced expansion [49,50].

Several chili pepper genes are highly expressed in flowers, such as CA08g18780, CA00g90800, CA00g90790, CA03g02050 and CA05g17060, indicating that type III PKS genes play an important function in flowers. However, little information is available on the flavonoid profile in chili pepper flowers. But it has been shown that flavonoids are highly accumulated in flowers in other plants, rendering flower colors [51,52]. Expression of only a portion of genes was detected across developmental stages of chili pepper leaves and fruits. Two Class II genes showed expression patterns in leaves and fruits which are consistent with the previously reported flavonoid accumulation patterns, indicating that CHS plays an important role in flavonoid biosynthesis in chili pepper [30,31].

In summary, we have identified 13, 14 and 13 type III PKS genes in *C. annuum*, *C. chinense* and *C. baccatum*, respectively, which can be classified into two classes. Genes in Class II likely encode CHS, which have experienced extensive expansions in *Capsicum* as well as in other Solanaceae species, and some of which are the result of tandem duplications, forming three clusters in the genome of *C. annuum*. Five genes are highly expressed in chili pepper flowers, as revealed by RNASeq data, which also show that the expression of two Class I genes was complementary to each other. Some of the Class II genes had upregulated expression as the chili pepper leaves matured and demonstrated downregulated expression with the maturation of fruits, consistent with flavonoid accumulation trends in chili pepper as reported previously. The results in this article will lay the foundation for future studies of type III PKS genes and the mechanisms of flavonoid biosynthesis in *Capsicum*.

## Appendix

**Table A1:** Locus IDs of type III PKS genes in three *Capsicum* species

Class	Clade	*C. annuum*	*C. chinense*	*C. baccatum*	Predicted activity
I		CA00g32570	CC.CCv1.2.scaffold703.5	CB.CBv1.2.scaffold2317.2	Sporopollenin synthase
		CA08g18780	CC.CCv1.2.scaffold968.30	CB.CBv1.2.scaffold411.22	
II	a		CC.CCv1.2.scaffold1329.2	CB.CBv1.2.scaffold400.45	Chalcone synthase
	b	CA00g90800	CC.CCv1.2.scaffold225.26	CB.CBv1.2.scaffold1096.23	
		CA00g90790	CC.CCv1.2.scaffold225.25	CB.CBv1.2.scaffold1096.24	
		CA00g90780	CC.CCv1.2.scaffold225.24	CB.CBv1.2.scaffold1096.25	
	c	CA12g20060	CC.CCv1.2.scaffold225.10	CB.CBv1.2.scaffold685.19	
		CA12g20070	CC.CCv1.2.scaffold225.12	CB.CBv1.2.scaffold685.20	
		CA12g20050	CC.CCv1.2.scaffold225.9		
		CA08g19650	CC.CCv1.2.scaffold694.6		
			CC.CCv1.2.scaffold225.13		
	d	CA03g02050	CC.CCv1.2.scaffold1326.8	CB.CBv1.2.scaffold1481.11	
	e	CA05g17060	CC.CCv1.2.scaffold717.24	CB.CBv1.2.scaffold400.10	
		CA05g17040	CC.CCv1.2.scaffold717.22	CB.CBv1.2.scaffold400.12	
		CA05g17030		CB.CBv1.2.scaffold400.11	
				CB.CBv1.2.scaffold400.13	
